# Current Practice of Public Involvement Activities in Biomedical Research and Innovation: A Systematic Qualitative Review

**DOI:** 10.1371/journal.pone.0113274

**Published:** 2014-12-03

**Authors:** Jonas Lander, Tobias Hainz, Irene Hirschberg, Daniel Strech

**Affiliations:** Institute for History, Ethics and Philosophy of Medicine, CELLS-Centre for Ethics and Law in the Life Science, Hannover Medical School, Carl-Neuberg-Street 1, 30625 Hannover, Germany; University Paris South, France

## Abstract

**Background:**

A recent report from the British Nuffield Council on Bioethics associated ‘emerging biotechnologies’ with a threefold challenge: 1) uncertainty about outcomes, 2) diverse public views on the values and implications attached to biotechnologies and 3) the possibility of creating radical changes regarding societal relations and practices. To address these challenges, leading international institutions stress the need for public involvement activities (PIAs). The objective of this study was to assess the state of PIA reports in the field of biomedical research.

**Methods:**

PIA reports were identified via a systematic literature search. Thematic text analysis was employed for data extraction.

**Results:**

After filtering, 35 public consultation and 11 public participation studies were included in this review. Analysis and synthesis of all 46 PIA studies resulted in 6 distinguishable PIA objectives and 37 corresponding PIA methods. Reports of outcome translation and PIA evaluation were found in 9 and 10 studies respectively (20% and 22%). The paper presents qualitative details.

**Discussion:**

The state of PIAs on biomedical research and innovation is characterized by a broad range of methods and awkward variation in the wording of objectives. Better comparability of PIAs might improve the translation of PIA findings into further policy development. PIA-specific reporting guidelines would help in this regard. The modest level of translation efforts is another pointer to the “deliberation to policy gap”. The results of this review could inform the design of new PIAs and future efforts to improve PIA comparability and outcome translation.

## Background

New developments in biomedical research, such as biobank-based research and gene transfer methods, as well as biomedical innovations such as synthetic biology, regenerative medicine, neuroimplants and nanotechnology, particularly attract public attention. In 2009, for example, biobanking was included by the Time Magazine in a list of “10 Ideas Changing the World Right Now” [1]. A recent report from the British Nuffield Council on Bioethics associated emerging biotechnologies with a threefold challenge that demands a more intensive “public discourse ethics”. The three challenges are: 1) “uncertainty” about outcomes; 2) “ambiguity”, meaning disagreement or diverse views and perceptions about the importance, values and implications attached to biotechnologies; and 3) the “transformative potential” to create large-scale, unexpected changes and disrupt existing technologies, relations and practices [2].

Further, specific social, ethical and legal challenges, such as the communication and commercialization of research results, the balancing of individual rights against the collective good, and data protection, to name but a few, require careful consideration and governance. This may be relevant not only to the approval and application of new biomedical technologies, but also to the processes of the underlying research [3]–[5].

To address these challenges, leading international institutions stress the importance of public involvement in biomedical research and innovation [2], [6]–[8]. Public involvement activities (PIAs) are often classified into (3–5) categories with different approaches and objectives, e.g. information/communication, consultation and participation/deliberation [6], [9]–[14].

For this paper, public information is understood as a *one-way* activity in which scientists provide the public with relevant information on a particular subject “to help them gain knowledge” [12] and ensure that the public can make informed decisions or arrive at an informed opinion. Common methods include websites, information events, and reading material [11]. Public consultation activities seek input from the public in order to consider (informed) public interests and opinions, for instance in policy development or prior to implementing a new technology. This type of PIA can be considered a one-way process too, in that the aim of the PIA ‘initiator’ (a scientific or policy actor) is to receive input from the public, for instance via focus groups, citizen councils or surveys; it is implied that decisions on the issue remain the responsibility of the initiator [9], [11], [12], [14]. Lastly, public participation/deliberation refers to some sort of reciprocal, informed dialogue between science/policy and the public; it differs from public consultation since the public is assigned a more active role not only in the dialogue but also in concomitant decision-making and policy development processes. Typical methods include advisory boards and juries including lay people/public representatives, consensus conferences, and dialogue sessions [2], [6], [11].

Previous studies have assessed and discussed the state of PIAs in health policy [15], [16], bioethics [17], health technology assessment [18], and in the development of research designs for primary health research [19] by using qualitative data analysis, “selective reviews” [15] and narrative review methods.

These previous studies have revealed that A) the majority of PIAs are carried out in the USA, Canada and the UK [15]; B) there is a considerable degree of diversity and lack of specificity regarding PIA objectives and methods [14], [15]; C) there is insufficient translation and evaluation [4], [5]; and D) evidence of the impact of PIAs is limited [13], [16], [20], [21].

The objective of this study was to assess a systematically-derived set of published PIAs in the field of biomedical research and innovation for A) their general characteristics (site, topic, duration and number of participants, year of conduct and year of publication); B) their study aims; C) their use at the planning stage of frameworks or findings from previous PIAs; D) the types of PIA method used; E) the translation of PIA outcomes to policy and practice; and F) measures used for PIA evaluation.

## Methods

### Database-specific search and selection of studies

We conducted a systematic literature search in the bibliographic databases PubMed, PsychInfo and Scopus from June 14 to July 29, 2013 with an update after three months. We restricted the search to studies published between January 2000 and June 2013 to account for the (most) recent developments in the field of biomedical research, in any language. The search queries combined comprehensive, general search terms representing “public involvement” and “research and innovation” in the field of biotechnology (Table 1). We chose very general and thus unspecific search queries because the indexing of PIAs in electronic databases is not yet well developed. We scanned all the located references for relevance by reading the title, abstract and keywords. Next, we read the full text of potentially relevant articles. We included a publication if it reported directly on the conduct and results of a PIA and if the focus of the PIA was on a topic in biotechnology research and innovation.

**Table 1:** Database-specific search queries used for the systematic search of publications of PIA reports.

**PubMed**
(((“biomedical research”[mesh]) OR (((((“Nanomedicine”[Mesh]) OR “Individualized Medicine”[Mesh]) OR “Genetic Therapy”[Mesh]) OR (((((“Biotechnology”[Mesh]) OR “Cell Engineering”[Mesh]) OR “Tissue Banks”[Mesh]) OR “Synthetic Biology”[Mesh]) OR “Regenerative Medicine”[Mesh]))))) AND ((((“Consumer Participation”) OR ((((((“public engagement”) OR “public participation”) OR “public involvement”) OR “public deliberation”) OR “public consultation”) OR “Consumer Participation”[Mesh]))))
**PsychInfo**
exp Involvement/or exp Community Involvement/or public involvement.mp. or exp Public Opinion/or public consultation.mp. or public engagement.mp. and exp Genetics/or exp Bioethics/or exp Informed Consent/or biomedical research.mp. or biotechnology.mp. or exp Biotechnology/
**Scopus**
TITLE-ABS-KEY(biotechnology) OR TITLE-ABS-KEY(“biomedical research”) AND TITLE-ABS-KEY(“public involvement”) OR TITLE-ABS-KEY(“public engagement”) OR TITLE-ABS-KEY(“public participation”) OR TITLE-ABS-KEY(“public consultation”)

### Data extraction and synthesis

We used a thematic text analysis approach for data extraction [22]. Data extraction used an assessment matrix listing core aspects of PIAs grouped under four main categories: A) general characteristics, B) PIA concept, C) background/theoretical foundation, and D) methods/procedures (Information S1). The core aspects were identified by a literature review (snowball sampling) of PIA-specific guidelines, frameworks, handbooks, manuals and studies [7], [9], [11], [12], [23]–[26].

All included studies were read in full and assessed independently by three researchers (JL read and assessed all, IH read and assessed 13 and TH read and assessed 33). According to thematic text analysis practice, all relevant text passages related to the core aspects were extracted. A code/keyword was assigned to each extracted text passage to allow for subsequent qualitative and quantitative analysis.

A sample of five studies was assessed by three authors (JL, TH, IH) to pilot-test the construct validity of the assessment matrix and the consistency of the extraction procedure. Results were compared and unclear or divergent coding was resolved after discussion with the fourth author (DS). The consistency of further extractions and codings was discussed in ongoing meetings and discussions between all four researchers.

Based on international categorization schemes, we broadly distinguished the included PIAs either as a public consultation or a public participation/deliberation activity [6], [7], [9], [11], [12], [24], [25], [27]. Because of the many different and sometimes inconsistent wordings we did not directly relate our coding to the self-classifications of included PIA studies (if there were any self-classifications). Instead, we based our classification on the objectives and methods described in the respective papers. We classified a PIA (including methods such as focus groups, surveys, questionnaires etc.) as a ‘public consultation’ if it aimed at gathering input from the public without actively including them in further decision-making or deliberation processes. We classified a PIA as ‘public participation/deliberation’ if it assigned the involved public a more active role in further decision-making or deliberation with decision makers, or offered other means to influence further policy development.

## Results

### General Information

**Figure 1:**
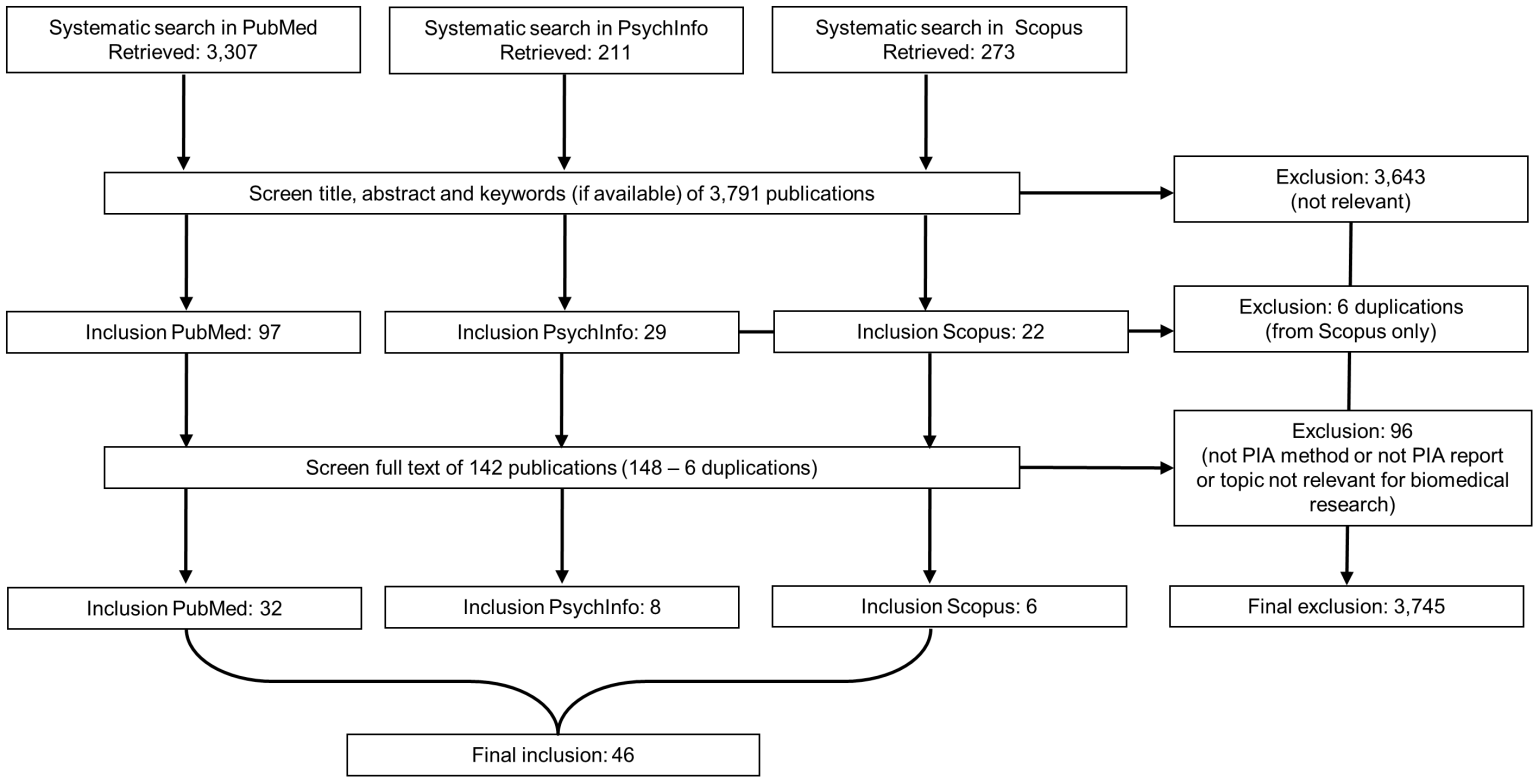
Selection of publications.

Of 3,791 publications identified via the comprehensive search, we finally included 46 PIA studies in our study (Fig. 1). Four studies discussed two similar PIAs but with distinct thematic foci [28]–[31].

Our studies were conducted in 15 different countries with 64% (n = 30) of studies being conducted in the USA (n = 18), Canada (n = 8) or Australia (n = 4). Those studies that provided a date (n = 39; 85%) were carried out between 1999 and 2011 and were published between 2002 and 2013 in 28 different journals (Information S2).

Genetic research was mentioned 20 times as the topic of the PIA (including genomics, pharmacogenomics and personalized genomics), and biobank research was mentioned 18 times (including biobanked specimens, DNA databases, genetic databases and DNA banks). The other topics were biomedical research in general (n = 5), research with bloodspot samples (n = 3), synthetic biology (n = 1), nanotechnology (n = 1), and human cloning research (n = 1). 28% of studies (n = 13) mentioned more than one topic.

Regarding the number of PIA participants, 35% of studies (n = 16) had fewer than 100 participants, 30% (n = 14) had 100–500 participants, and 35% (n = 16) had more than 500 participants.

24% of studies (n = 11) did not specify a duration. Of those that did (n = 35), 67% (n = 31) took longer than a day and 9% (n = 4) took one day or less (Table 2). 63% (n = 22) of those that reported a duration mentioned this only implicitly or used more general wordings such as “two separate weekends” [32] or “34 groups were involved in 50 discussions” [33].

**Table 2:** General information about the selected studies ordered by PIA category (public consultation and public participation/deliberation) and year of publication (descending); Ca./i., Circa/implicit, respective information was not reported clearly, i.e. reported with some ambiguity; N.S., not specified in PIA report; min, minute(s); h, hours.

Reference	Country	Topic(s)	Duration	Participants	Conduct	Publication
Public Consultation						
[39]	USA	Genetic research, biobanking	n.s.	1,041 (2 phases)	2009, 2011	2013
[40]	Canada	Biomedical research	n.s.	2,604	2010	2013
[41]	USA	Biomedical research	25 min (by 117 participants)	117	2009	2013
[42]	USA	Biobank-based research	16 h (8 groups by 2 hours)	45	n.s.	2013
[43]	USA	Genomic research	Ca. 48 min (by 91 participants)	91	n.s.	2013
[44]	Jordan	Biobanking	3 months (i)	3,196	2011	2013
[45]	USA	Biomedical research with newborn blood samples	9 months (i)	3,855	2010	2012
[46]	Canada	Health-related research with biobanked specimens	2 months (i)	330	2010	2012
[47]	USA	Genetics/genomics research	2 h (by 8 groups)	199	2009, 2010	2012
[48]	Austria	Synthetic biology, biotechnology, nanotechnology	n.s.	49	2008	2012
[49]	USA	Newborn blood sample research, genetic testing	6 months	128	2010	2012
[50]	Australia	Biobanking	n.s.	1,000	n.s.	2011
[51]	Sweden	Biobanking	7 min (by 23 interviews)	926	2007–2008	2010
[52]	Saudia Arabia	Medical research, tissue research	9 months (i)	528	2006–2008	2010
[53]	Egypt	Blood samples, genetic research	7 months (i)	600	2007	2010
[54]	UK	Genomic science	3 phases (Jul–Sept; Mar–Apr; Aug–Sept); 5–9 min (films); n.s. (interviews)	4,595 (3 phases)	2003–2004	2010
[55]	Finland	Biobanking	n.s.	1,195	2007	2009
[56]	USA	Pediatric samples, biobanks	30 min (by 1186 participant interviews)	1,186	2002–2003	2009
[57]	Canada	Genetic database	n.s.	Ca. 4,017 (5 phases)	2001, 2003	2009
[58]	Japan	Pharmacogenomics research, DNA bank, Genomic markers	2 months (i)	550	2008–2009	2009
[31]	USA	Biobanking	2 h (by 15 groups)	60	2007–2008	2009
[59]	USA	Personal genomics	n.s.	396	2009, 2010	2008
[60]	Malawi	Biomedical research studies/Health research studies	n.s.	108	n.s.	2008
[28]	USA	Genomic biobanking	2 h (by 15 groups)	Ca. 60	2007	2008
[36]	Scotland	DNA database	n.s.	Ca. 17	2003–2004	2008
[61]	USA	Biomedical research	3 months (i)	900	2003	2007
[62]	UK	Human cloning (research)	3 phases: 4 months, 5 months, 100 months (i)	Ca. 2,960	2003	2007
[63]	Canada	Genomics research and biobanking	n.s.	11	n.s.	2007
[64]	USA	Genetics research	2 h (by 63 participants)	63	n.s.	2005
[33]	Kenya	Biomedical research	1–2 h (by 34 groups)	Ca. 270	2001–2002	2005
[65]	USA	Genetics	8 months	215	2001–2002	2005
[66]	USA	Genetic research	2 h (by 9 groups)	91	2002	2004
[67]	Singapore	Genetic research	2 h (by 12 groups)	98	2002	2004
[68]	n.s.	Disease/genetic susceptibility research	1 h (by 37 participants)	37	n.s.	2003
[69]	Germany	Pre/postnatal genetic testing	n.s.	2,076	2001	2002
Public Participation/Deliberation						
[70]	Kenya	Genetic/genomic research	11 months (i)	63	2009–2010	2013
[71]	Canada	Research with bloodspot samples from newborn screening	n.s.	60	2009	2012
[30]	Australia	Biobanking, health policy for biobanking	4 days	16	2008	2012
[32]	Canada	Biobanking, institutional biobanking policy	4 days (2 weekends)	25	2009	2012
[72]	USA	Genetic variation and happlotype mapping	2 h+1.5 h+1 day	More than 250 (3 phases)	2003–2004	2012
[29]	Australia	Biobanking	4 days	17	2008	2011
[73]	Canada	Health Technology Assessment	2.5 days	420 (survey); 16 (jury)	n.s.	2008
[74]	Canada	Biobanking	2 weekends	21	2007	2008
[75]	Australia	Clinical genetics services	n.s.	Ca. 400	2005	2008
[76]	USA	Personalized-medicine research project, biobanking	n.s.	144 (4 phases)	2001 (i)	2008
[77]	USA	Genetic research and technology	6 months (i)	63	1999	2003

### Study objectives

In the 46 PIAs we found 66 different (wordings for) objectives, such as “assess the public's perceptions”, “offer collective responses that should be understood as tailored policy input” or “determining the feasibility of conducting a citizens [sic] jury”. These different wordings were grouped under 6 distinguishable PIA objectives (see Table 3).

**Table 3:** PIA Objectives and Methods.

Objectives	Text Examples	Count (n)	Methods related to the PIA objectives	Count (n)
**a)** Inform/educate (prior to consultation or participation/deliberation)	The forum had four broad objectives: (i) Inform a representative sample of citizens of the competing interests and perspectives on biobanking, […]	2	Long film	1
			Short film	6
			Informational/introductory presentation	10
			Literature (booklet, pamphlet, etc.)	11
			Showcards	2
	The overall goal of this component of the consultation and communication plan was to educate residents of the 19 Zip code region about the PMRP through talks to community groups and media prior to enrollment, and then through regular newsletters after study participation.		Workbook (to guide group discussion)	1
			Presentation of newspaper article	1
			Vignettes (Description of current studies/standardized scenarios)	2
			Media release (to announce project)	1
			Blog (online)	1
**b)** Consult the public to gather attitudes, opinions, preferences, etc. either	To assess the general attitudes towards genetic research and participation in biobanks in the Long Island/Queens area of New York, and what factors would predict a positive view of such research, participants from the NSLIJ hospital system were surveyed.	44	**c.1)** …without further discussion:	
• as a means to use this input for further policy development/decision-making without subsequent active engagement of the consulted public			Questionnaire	11
• or in advance of/as part of a public engagement activity			Questionnaire guide	1
			Written Survey	16
			Telephone survey	3
			Online survey	3
			Phone interview	3
			Face to face interview	11
			Email questions	1
			Ranking of research scenarios (by participants)	3
			Discrete choice experiment	1
	To assess the public's perception of biobank research and the relative importance they place on concerns for privacy and confidentiality, when compared with other key variables when considering participation in biobank research.		Interactive discussion game	1
	We explored Canadian values regarding storage and use of NBS samples for various purposes and the forms of parental choice for anonymous research with NBS samples.		**c.2)**…with further discussion:	
			Focus Group	18
			Group deliberation/dialogue	5
			Small group discussion/deliberation	6
			Large group discussion/deliberation	4
			Feedback Session	1
			Panel interaction	1
			Citizen jury (covers c2 and d1)	1
			Moderator guide (guiding focus groups)	3
			Ratification process (by group, of recommendations/resolutions)	1
**c)** Engage the public actively in policy development/decision-making processes	[…] because the purpose of the research was to inform policy development within NBS and an overview of all of the categories provides guidance on these issues.	11	**d.1)** Disseminating/translating PIA outcomes:	
			Dissemination via print and electronic media	4
			Recommendation report (written)	4
	In a significant refinement of methods, we focus on providing public input to institutional practice and governance of biobanks using a tailored workbook structure to guide participants' discussion.		One-day conference	1
			Group recommendations (spoken)	2
			Community project oversight, project advisory	3
	The results of this deliberation offer collective responses that should be understood as tailored policy input, rather than public opinion measurement.		Meetings to discuss results	2
			**d.2)** Considering PIA outcomes in policy:	
			Decision-maker response/feedback (to deliberant's recommendations)	1
**d)** Investigate impact of PIA on participants	In a real-world experiment, this study on synthetic biology investigated the effect of information uptake and deliberation on opinion certainty and opinion valence in natural groups.	4	See c.1) and c.2) for methods	
	As part of our deliberative engagement, we surveyed the participants both before and after the engagement intervention to determine whether there were attitudinal changes.			
	The purpose of this paper is to explore whether members of the public recall TSUS and whether they use the study to interpret current biomedical research.			
**e)** Describe PIA method	In response to this gap in the literature (and to address the deliberative norms of transparency and publicity), the purpose of our paper is to […] describe the processes by which we translated these outputs to policy.	2	n.a.	
	The purpose of this paper is to illustrate a novel method for developing meaningful public input on ethically contentious issues in institutional biobanking policy.			
**f)** Test PIA method	The main goals of the PEGV Project were to test a community engagement model, […]	6	See a)–d) for methods (the respective PIA method was tested by running the individual methods described above, for instance by handing out reading material to participants)	
	[…] 2 determining the feasibility of conducting a citizens [sic] jury to elicit the views of the public on priorities for HTA;			

62% of objectives (n = 42) related to public consultation, i.e. to a broad understanding of ‘attitudes research’, for example, “to assess attitudes”, “to inquire perceptions [sic]” or “to query about preferences [sic]”.

16% of objectives (n = 11) related to public participation/deliberation, for instance by offering “collective responses that should be understood as tailored policy input, rather than public opinion measurement”.

3% of 68 objectives (n = 2) related to public information. Since our analysis did not include pure public information activities (that is, activities with no further means of public consultation or participation), these objectives were ‘only’ sub-objectives within two PIAs considered as public participation/deliberation (see the Methods section).

The remaining objectives related to a broad understanding of and contribution to PIA research, i.e. describing a PIA method (n = 2), investigating the impact of the PIA on participants (n = 3), and testing a PIA method (n = 6). Overall, 20% (n = 9) of all PIAs reported objectives related to PIA research (two PIAs reported more than one research objective).

### PIA categories and PIA methods

We rated 76% (n = 35) of the studies as public consultations, and 24% (n = 11) as public participations/deliberations. In total, we found 146 wordings for the methods applied in the 46 PIAs. However, these wordings often described similar methods and therefore were grouped according to the 6 PIA objectives (see Table 3). Within these 6 PIA objectives, we distinguished 37 individual methods, such as short video (n = 6), literature (n = 11), written survey (n = 16), and citizen jury (n = 1).

‘Public information’ contained many more methods (n = 36) than corresponding objectives (n = 2). This is due to the fact that several PIAs (n = 23) reported the use of informational material such as literature or introductory presentations without also explicitly stating the objective of providing information prior to consultation or participation/deliberation.

### References to previous PIA reports, previous PIA research, or PIA guidelines

93% (n = 43) of all studies referred to other PIA reports or PIA research. 42% (n = 18) of all studies explicitly used these references as the methodological framework for their own PIA or to validate their choice of PIA methods. Three studies did not refer to any methodology.

Furthermore, no study reported the explicit or implicit use of public involvement guidelines that describe different PIA objectives and methods (such as “Planning guide for public engagement and outreach in nanotechnology” [7]).

### Evaluation and translation of findings

22% of all studies (n = 10) reported some evaluation measures, which related to A) participants' satisfaction during participation (n = 6) or attitude changes (n = 2) and/or B) effectiveness and feasibility of the PIA method (n = 4); two PIAs reported both A) and B).

One of the 35 public consultation studies (3%) and 8 of the 11 public participation/deliberation studies (73%) reported some sort of translational efforts to either disseminate study findings and recommendations or communicate them directly to decision-makers in policy and practice. Translational efforts included A) a final conference and (spoken) presentation of recommendations (n = 3); B) the writing and dissemination of a policy-oriented final report (n = 4); C) community guidance during and after the PIA (n = 3); D) dissemination via print and electronic media (n = 4); and E) dialogue-oriented meetings between researchers and politicians to discuss results (n = 2).

## Discussion

This study assessed a systematically-derived sample of 46 peer-reviewed journal articles reporting on public involvement activities (PIAs) in the field of biomedical research and innovation. The 35 public consultation and 11 public deliberation/participation activities used a broad range of different wordings for PIA objectives and corresponding methods. Our categorization reduced this range to 6 different PIA objectives and 37 corresponding PIA methods. These findings confirm previous reviews of PIAs in other areas such as health policy, concluding that public involvement as a tool for governance and democratic decision-making is defined and practiced in many ways [2], [10], [15], [34].

Regarding the clustered PIA objectives in particular, it emerged that a range of significant, classical objectives such as building public trust, empowering individuals to actively engage in political debate, and legitimizing policy were not explicitly stated as objectives in the respective PIA reports [35]. Rather, these objectives were addressed in the study's background sections in a more general sense, e.g. “In the UK, development of DNA databases […] is set against a perceived ‘public crisis in trust’ in both medical and scientific spheres, which, it is argued, has set up ‘a new mood for dialogue’ within the science community […] as a way to boost confidence and reinstate trust” [36]. The lack of objectives related to public trust, empowerment, legitimacy, etc. may partly be explained by the difficulty in linking such ‘thick normative concepts’ like trust to one particular PIA method. In scientific papers, however, the ‘primary’ objectives are directly linked to a specific method. One core task in peer-reviewing scientific papers is to check whether the stated objectives match the methods employed. For instance, the objective of gathering the public's views may be achieved via a focus group, and the objective of policy translation may be achieved via a public hearing based on the focus group findings; but the overarching aim of increasing public trust in a certain biomedical research process does not straightforwardly suggest how to achieve this. Despite these difficulties it might be important to report explicitly what the underlying normative rationale for performing a certain PIA was. This information might support better interpretation of PIA reports by their readers.

Though there is relatively established terminology for PIA categories and objectives [2], [11], [12] as well as guidelines for designing PIAs [6], [7], [9], [24], [25], [27], neither was considered in any of the 46 papers included in this review. While a proliferation of methods should not be seen problematic in itself, easier comparability of PIAs for biomedical research and innovation might improve the translation of PIA findings into further policy development. The development of reporting standards and best practice examples for PIA objectives and methods could be a first step in this direction. The EQUATOR network (www.equator-network.org) might be a good partner for starting the development of such reporting standards.

While research into the quality of PIAs on biomedical issues is important to further strengthen the validity and practical relevance of the public involvement approach, it is currently not widespread. Only 10 (22%) of the identified PIAs reported on evaluation measures such as participants' satisfaction with their participation, changes in attitudes as a result of participation, and the effectiveness and feasibility of PIA methods.

Only 9 (20%) of the 46 PIA studies reported on translation of their findings, despite the importance attributed to this aspect of PIAs [2], [4], [30] The rather modest translation efforts found in this analysis confirm the “deliberation to policy gap” described in other fields of public involvement [4]. Recent reports on public involvement in patient and health services suggest that the translation of findings of PIAs initiated by academic institutions/departments is often limited to publication in peer-reviewed journals, since this is often seen as the main means of research translation [37]. As the PIAs in this review were solely initiated by academic departments, this may partly explain the low use (or at least low reporting) of translation methods. In this sense it is also suggested that “the relationship between the hosts of a public engagement and institutional bodies who actually make policy on the issues under consideration clearly has an impact on the mandate of the public forum to influence policy” [4]. Hence, a potential translation challenge may arise when an (academic) PIA initiator lacks a strong relationship with the (political) body to which results should be translated. Such potential ‘distance’, i.e. divergent nature of work, methods, thematic priorities etc. point to a further aspect that could be considered important for the translation of findings: for PIAs to be relevant for policy development in a particular area, the PIA's topic and outcomes need to account for institutional/policy contexts, rather than only for the ‘research context’ [16], [38]. This means for instance that PIA findings need to be accompanied by practical recommendations on issues that can actually be addressed at a political level. Overall, translation of PIA results is important not only to answer questions for policy, but also questions that improve public understanding of biomedical research (for example regarding data protection in biobanks), i.e. questions of the “public good” [2].

Regarding the analysis of the aspect of translation, we stress the limitation that our analysis only assessed whether translation efforts were reported or not. It might be that translation of PIA findings is often practiced but seldom reported in scientific journals. This may be true especially for public consultation activities, which are not necessarily limited to merely gathering public opinion. However, if the findings of public consultation activities are in fact used to influence policy, future PIA reports could address this more explicitly. In order to improve insight into challenges for translation of consultation and participation/deliberation activities, further research could assess the importance policy and decision-makers actually place on PIA findings. In this sense, it could also be assessed whether there are sufficient ‘contact points’ in policy or practice for communicating PIA findings.

Despite the fact that we employed narrow as well as general search terms for the broad field of biomedical research and innovation, most of the identified PIAs dealt with genetics or biobanking. The fact that biobanking and genetics (potentially) concern more of the public than other emerging biotechnologies such as gene therapy, nanotechnology, neuroimplants, tissue engineering and synthetic biology may partly explain this gap. With recent calls for more public involvement in the field of biomedical research, more PIAs on issues beyond genetics and biobanking are to be expected. The design of new PIAs on relevant issues in specific disciplines of biomedical research could be informed by the results of this review.

We highlight that our categorizations and classifications of text passages from the included PIA reports involved interpretation. We addressed this challenge by having at least two researchers analyzing each text independently, discussing differences and finally agreeing classifications among all four authors. Also, PIAs conducted in disciplines other than biomedical research as well as PIAs published in formats other than peer-reviewed scientific journals may suggest different reporting practices, for instance regarding details on ‘direct’ objectives (to consult the public) and more indirect ones (to increase public trust).

Further consideration of existing PIA guidelines and the development of PIA-specific reporting guidelines could improve the comparability of PIAs, streamline their reporting and thus enforce the still lacking (or underreported) translation of findings.

## Supporting Information

Checklist S1 **PRISMA Checklist.**
(PDF)

Information S1 **Assessment matrix.** The matrix used to analyze selected studies is divided according to the core aspects of Public Involvement Activities identified by a literature review.
(PDF)

Information S2 **Overview of journals that published selected PIA reports.** Sorted according to (1) the number of studies selected from the respective journal and (2) alphabetical order.
(PDF)

Information S3 **Electronic search strategy.**
(PDF)
