# Supplementary material for: Current Practice of Public Involvement Activities in Biomedical Research and Innovation: A Systematic Qualitative Review
Source: PLoS One. 2014 Dec 3;9(12):e113274. doi: 10.1371/journal.pone.0113274 (PMC4254603; doi:10.1371/journal.pone.0113274)
Supplement: Information S1 — Assessment matrix. The matrix used to analyze selected studies is divided according to the core aspects of Public Involvement Activities identified by a literature review. (PDF) [file pone.0113274.s002.pdf]

Supporting Information 1

Assessment matrix. The matrix used to analyze selected studies is structured according to the core aspects of Public Involvement Activities identified by a literature review.

**Item**	**Item specification (if relevant)**	**Original text passage**	**Keyword / Code**
Reference			
**A) General Characteristics**			
Topic			
Year of PIA conduct			
Year of Publication	Online first		
Methodological	a) Duration		
	b) Study participants (N)		
Country			
Journal			
**B) PIA Concept**			
PIA Category	a) consultation		
	b) deliberation-participation		
Method(s)			
Study objective(s)			
**C) Background / Theoretical Framework**			
Reference to previous PIA report / PIA research / guideline / other			
	a) Previous PIA report		
	b) PIA research		
	c) PIA guideline		
	d) Other		
**D) Past PIA conduct**			
Translation			
Evaluation	a) Evaluation of PIA methodology by PIA researchers		
	b) Feedback from study participants		
Limitations	Regarding PIA conduct		
