# Supplementary material for: Current Practice of Public Involvement Activities in Biomedical Research and Innovation: A Systematic Qualitative Review
Source: PLoS One. 2014 Dec 3;9(12):e113274. doi: 10.1371/journal.pone.0113274 (PMC4254603; doi:10.1371/journal.pone.0113274)
Supplement: Information S2 — Overview of journals that published selected PIA reports. Sorted according to (1) the number of studies selected from the respective journal and (2) alphabetical order. (PDF) [file pone.0113274.s003.pdf]

Supporting Information 2

Overview of journals that published selected PIA reports. Sorted according to (1) the number of studies selected from the respective journal and (2) alphabetical order.

Journal	PIA reports (n)
Social Science and Medicine	7
Public Understanding of Science	4
American Journal of Medical Genetics	3
Journal of Medical Ethics	3
Public Health Genomics	3
Community Genetics	2
Genetics in Medicine	2
Health Expectations	2
Journal of the National Medical Association	2
Pediatrics	2
Academic Medicince	1
American Journal of Bioethics	1
American Journal of Health Behaviour	1
American Journal of Public Health	1
Biopreservation and Biobanking	1
BMC Medical Ethics	1
BMC Public Health	1
Cell Tissue Bank	1
Comprehensive Psychiatry	1
Critical Public Health	1
Ethik in der Medizin	1
Journal of Assisted Reproduction and Genetics	1
Journal of Pediatrics	1
Malawi Medical Journal	1
North Carolina Medical Journal	1
Population Health Management	1
Scandinavian Journal of Public Health	1
Zeitschrift für Gesundheitspsychologie	1
