# Supplementary material for: Current Practice of Public Involvement Activities in Biomedical Research and Innovation: A Systematic Qualitative Review
Source: PLoS One. 2014 Dec 3;9(12):e113274. doi: 10.1371/journal.pone.0113274 (PMC4254603; doi:10.1371/journal.pone.0113274)
Supplement: Information S3 — Electronic search strategy. (PDF) [file pone.0113274.s004.pdf]

Supporting Information 3

Search strategy for search in PubMed

Step 1

((("biomedical research"[mesh]) OR ((((("Nanomedicine"[Mesh]) OR "Individualized Medicine"[Mesh]) OR "Genetic Therapy"[Mesh]) OR ((((("Biotechnology"[Mesh]) OR "Cell Engineering"[Mesh]) OR "Tissue Banks"[Mesh]) OR "Synthetic Biology"[Mesh]) OR "Regenerative Medicine"[Mesh])))))

Step 2

(((("Consumer Participation") OR (((((("public engagement") OR "public participation") OR "public involvement") OR "public deliberation") OR "public consultation") OR "Consumer Participation"[Mesh]))))

Step 1 and Step 2
